# Evaluating and Mapping Grape Color Using Image-Based Phenotyping

**DOI:** 10.34133/2020/8086309

**Published:** 2020-04-24

**Authors:** A. N. Underhill, C. D. Hirsch, M. D. Clark

**Affiliations:** ^1^Department of Horticultural Science, University of Minnesota, St. Paul, MN, USA; ^2^Department of Plant Pathology, University of Minnesota, St. Paul, MN, USA

## Abstract

Grape berry color is an economically important trait that is controlled by two major genes influencing anthocyanin synthesis in the skin. Color is often described qualitatively using six major categories; however, this is a subjective rating that often fails to describe variation within these six classes. To investigate minor genes influencing berry color, image analysis was used to quantify berry color using different color spaces. An image analysis pipeline was developed and utilized to quantify color in a segregating hybrid wine grape population across two years. Images were collected from grape clusters immediately after harvest and segmented by color to determine the red, green, and blue (RGB); hue, saturation, and intensity (HSI); and lightness, red-green, and blue-yellow values (L∗a∗b∗) of berries. QTL analysis identified known major QTL for color on chromosome 2 along with several previously unreported smaller-effect QTL on chromosomes 1, 5, 6, 7, 10, 15, 18, and 19. This study demonstrated the ability of an image analysis phenotyping system to characterize berry color and to more effectively capture variability within a population and identify genetic regions of interest.

## 1. Introduction

The skin and flesh color of grape (*Vitis* spp.) berries are important traits that have a large impact on the end use of the fruit. In both wine and table grapes, fruit color is a critical breeding target; both noir (“red,” “blue,” or “black”) and nonnoir (“green” or “white”) grapes can be desirable, depending on the intended use. In table grapes, berry color has been shown to impact consumer preference [1], while wine grape color influences the color of the final wine. Berry color is the visual manifestation of organic compounds known as anthocyanins within the skin and occasionally flesh, as is the case with *teinturier* (French for “to dye” or “to stain”) grapes. Responsible for blue, red, and purple colors, five anthocyanins are found in *Vitis* species: malvidin, petunidin, peonidin, cyanidin, and delphinidin [2]. These compounds exist in mono- and diglucoside forms along with acylated and nonacylated forms, and the proportions of each type found in fruit can be used to differentiate species [3].

Inheritance of color is neither purely qualitative nor purely quantitative: though color class (noir vs. nonnoir) is inherited in a Mendelian fashion, other elements of color determination, like anthocyanin concentration, seem to be quantitative and under environmental control. The two-gene model of color inheritance is currently supported by molecular evidence, and advances in marker technologies have allowed the elucidation and mapping of the entire anthocyanin pathway. Several genes have been identified as playing a role in color determination; nearly all of them code for enzymes in the anthocyanin production pathway. *PAL*, *CHS*, *CHI*, *F3H*, *F3'H, F3'5'H*, and *DFR* are each responsible for the synthesis of a different precursor in the pathway [4–7]. *LDOX* creates the anthocyanins cyanidin and delphinidin from leucocyanidin and leucodelphinidin through oxidation [6], and *AOMT* methylates these anthocyanins to create peonidin, petunidin, and malvidin [8]. Two genes not involved in the pathway itself are *VvMYBA1* and *VvMYBA2*, both of which are transcription factors responsible for regulation of the anthocyanin pathway by controlling *UFGT* [9]. Although color variation is largely explained by the haplotype of these two genes, other variables are still influential. Different alleles can be present in the diverse germplasm, and unique color haplotypes exist after a history of mutation and human selection in grape [10]. Combinations of different haplotypes are one factor in color expression and anthocyanin differences between cultivars, yet some cultivars do not exhibit the expected color based on their haplotype [11]. Environmental effects on anthocyanin synthesis have been well-documented. Temperature [12], sun or shade exposure [13], and plant hormones [14, 15] all contribute to accumulation or degradation of anthocyanins. Additionally, pH increases during ripening and differing total acidity levels among cultivars impact the visual color of anthocyanins [16]. Ultimately, although the question of genetic color determination in grapes is well-understood, the exact biology of final berry color is complex.

Describing the berry color phenotype can be done in multiple ways. Some methods are as simple as classifying fruit as noir or nonnoir, while others classify fruit qualitatively based on perceived hue. Example color classes are described by International Organisation of Vine and Wine (L'Organisation Internationale de la Vigne et du Vin, OIV) descriptor code 225, where berries are classified as either green yellow, rose, red, grey, dark red violet, or blue black [17]. However, other methods are more quantitative: for example, measuring the amount of anthocyanin in fruit is not an exact measure of color itself but provides insight into the chemical makeup resulting in the final visual color. Quantifying anthocyanin content may be precise, but it is a time-intensive process requiring multiple steps and the use of specialized high-pressure liquid chromatography (HPLC) or spectrophotometry equipment. Hyperspectral imaging has been tested as a nondestructive alternative to HPLC, with relatively high prediction accuracy; however, this requires the use of expensive hyperspectral cameras [18]. In contrast, evaluating fruit using visual rating scales is quick, inexpensive, and relatively simple, but the process is difficult when dealing with fruit that is intermediately colored. For example, berries somewhere between rose and red are difficult to distinguish even to an experienced evaluator. Additionally, environmental variation leads to differences within a cluster which makes assigning a single score difficult, as sun-exposed berries can be several shades or even tones darker than those shaded by the canopy [19]. Some visual-based systems use a combination of quantitative and qualitative data to measure fruit color, such as the system used by Carreño et al. in which a chroma meter was used to measure the lightness (L∗), green-red value (a∗), blue-yellow value (b∗), hue angle, and chroma values of red table grapes [20]. Coupled with a visual assessment of fruit color, an index (Color Index for Red Grapes, CIRG) based on these measurements was proposed to delineate color classes (yellow-green, pink, red, violet, and dark violet) by numerical value; however, this value itself was not used for analysis. Image analysis systems on a field-wide scale have been used to classify fruit color both as a trait of interest [21] and as a proxy for the fruit development stage [22]. A quick, technologically simple way to collect precise data would be ideal for berry color.

When color information is to be used in genetic mapping experiments, capturing a large proportion of the population variance is key. Separating berry colors into two noir/nonnoir or six OIV classes may not be adequate, and minor genes related to color variation may not be detected in the mapping process. Evaluation based on a noir vs. nonnoir scale clearly shows Mendelian inheritance for berry color, and grouping berries into two classes (either “white, yellow, green, and amber” or “red and black”) led early researchers to expected segregation ratios [23]. Additionally, experiments mapping color-related traits have been able to identify the major *VvMYBA* locus on chromosome 2 when using anthocyanin content, color class, or anthocyanin-related gene expression as a trait [24–27]. However, a recent study found many minor quantitative trait loci (QTL) for anthocyanins on several chromosomes, suggesting that regulation of berry color may extend beyond the two major loci [24]. More precise methods of visual color quantification will help to elucidate the finer color regulation mechanisms and aid in understanding the extent to which environment influences color in grape berries. Additionally, automated quantification can be used in many contexts: for example, more accurate descriptions of color across a range of *Vitis* species, such as those found in a germplasm repository. The ability to identify subtle differences in color could allow association mapping studies to be carried out in populations that are not segregating for color as measured by OIV descriptor 225 (for example, clusters that are all rated as rose or red). Image-based color evaluation can be used to compare color change over time between genotypes or to compare responses to color-influencing environmental or chemical factors. Coupling chemical data with visual color could help uncover relationships between the two and could potentially be used in lieu of more time-intensive evaluation techniques. Overall, a better understanding of berry color, led by improved phenotyping techniques, will help grape breeders target desirable color profiles and breed for them more efficiently.

In this study, images of grape clusters were evaluated for berry color using several color spaces (RGB, L∗a∗b∗, and HSI) to determine the most suitable for characterizing fruit. This data was used in QTL mapping in order to identify loci of interest that could be contributing to variation in fruit color.

## 2. Materials and Methods

### 2.1. Experimental Design

#### 2.1.1. Plant Material

Data were collected from a biparental F1 mapping population, GE1025, that was established from a cross (MN1264×MN1246) made in 2010. Both parents are breeding selections in the University of Minnesota wine grape program; their pedigree is detailed in Teh et al. [28]. This cross has diverse parentage, including ancestry in at least six *Vitis* species (*V. vinifera*, *V. riparia*, *V. rupestris*, *V. labrusca*, *V. aestivalis*, and *V. berlandieri*). Vines are maintained at the University of Minnesota Horticultural Research Center (44°52′08.1^″^N 93°38′17.3^″^W) and trained in a top wire, high-cordon system. MN1264 has noir-skinned berries (rating of 6, OIV descriptor 225), while MN1246 has nonnoir berries with rose to red color (rating of 2 to 3, OIV descriptor 225).

Data was collected from clusters produced in 2017 and 2018. At harvest (°Bx > 20; September 21-22 in 2017, August 29-30 in 2018), three representative fruit clusters were taken from each fruiting vine in the field (*n* = 121 in 2017 and *n* = 127 in 2018). Clusters were carefully removed from shoots so that the entire length of the peduncle was preserved. After harvest, fruit was transported immediately to the laboratory for image collection.

#### 2.1.2. Image Collection

**Figure 1:**
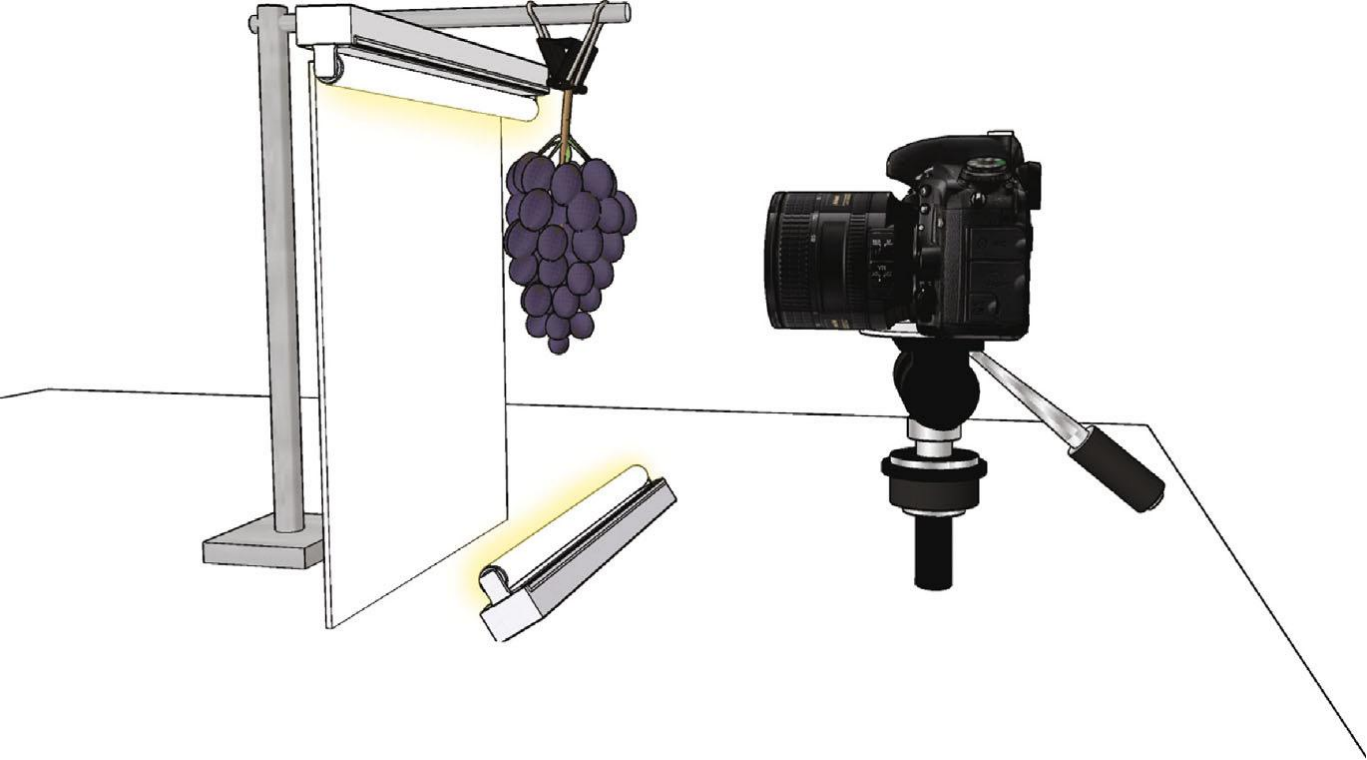
A diagram of the camera, lighting, and backdrop setup used for image capture (not to scale).

In a controlled setting, each cluster was suspended by its rachis using an alligator clip against a white backdrop. Four images were taken of each cluster with 90° rotations between each image, resulting in twelve images per genotype. Two fluorescent lights (Philips 34 W, 3500 K, Philips Lighting, Somerset, NJ, USA) were used to illuminate the fruit, and a tripod-mounted Nikon D7200 camera (24.2 megapixels) with a fixed 50 mm lens (Nikon, Tokyo, Japan) was used to capture images with the lens of the camera positioned 48 cm away from the rachis of the fruit. This design was based on a previously published imaging setup [29], and a diagram is provided in Figure 1.

**Figure 2:**
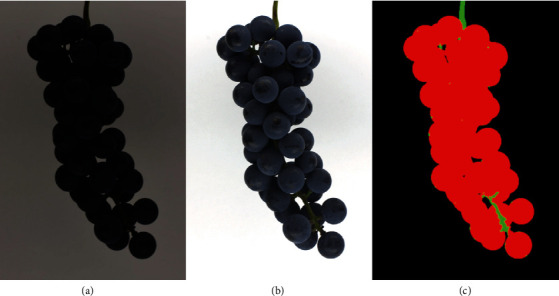
An example of a single genotype with raw (a), color-corrected (b), and segmented (c) cluster images.

The open source software package gPhoto2 (http://www.gphoto.org/), a command-line application for Unix-like systems, was used for image capture, file download, and file naming. Images were captured in Nikon RAW format (.nef; Figure 2(a)). An X-Rite ColorChecker Classic color card (Grand Rapids, MI, USA) and the software package RawTherapee (https://rawtherapee.com/) were used to ensure correct white balance and color in images; this adjustment was made by changing the temperature to 3395 and lowering the tint to 0.777 (Figure 2(b)). Images were batch processed for color corrections, then compressed and exported as 16-bit Tagged Image File Format (.tiff) files.

#### 2.1.3. Image Segmentation

Images were segmented into berry, rachis, and background components by color using the Food Color Inspector software (http://www.cofilab.com/portfolio/food-color-inspector/). This semiautomated method requires the definition of an initial training set, with the color of each component being selected by the user. After the training set is established, it can be used to process multiple images of the same individual or genotype, depending on the intragenotype or interindividual variation. Training sets were constructed manually using one image from a genotype, with 3 × 3 squares of pixels representative of berry, rachis, and background classes isolated by hand. With noir (blue- to black-colored) clusters, only one 3 × 3 pixel area selection was used to train each class. With nonnoir (light-colored) clusters, multiple 3 × 3 pixel areas were used to train each class; additional berry and rachis class selections were made until the segmentation was as accurate as possible. After a training set was constructed, the remaining images from that genotype were segmented automatically using the same training set. Images were then visually inspected for segmentation accuracy. If significant missegmentation was observed using a single training set, each image was processed again with its own training set. Segmented images were then exported as 24-bit Portable Network Graphics (.png) files (Figure 2(c)).

#### 2.1.4. Image Data Extraction

**Figure 3:**
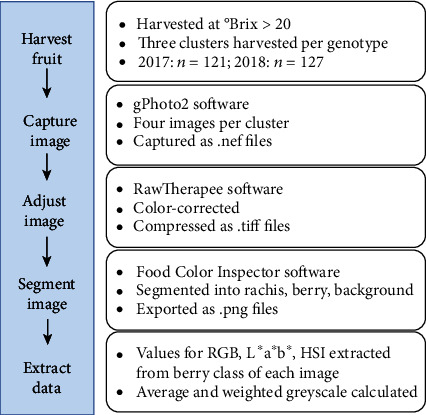
A flowchart of the image capture, segmentation, and data extraction process.

The red (R), green (G), blue (B), lightness (L∗), green-red (a∗), blue-yellow (b∗), hue (H), saturation (S), and intensity (I) values of the berry color class were then extracted from each segmented photo. Average greyscale and weighted greyscale values were calculated, where the average greyscale was equal to the mean of the R, G, and B values for each image, and the weighted greyscale was calculated using the relative luminance function with a red weight of 0.299, a green weight of 0.587, and a blue weight of 0.114. Additionally, cluster color was scored visually using OIV descriptor no. 225 [15] and sorted into binary noir/nonnoir categories. A flowchart of the image capture, segmentation, and data extraction process can be seen in Figure 3.

### 2.2. Statistical Analysis

Data from 121 individuals in 2017 and 127 individuals in 2018 were analyzed using R (https://www.r-project.org/). Component color values for each color space were averaged, giving one average value from twelve images of that genotype. Normality of color data was assessed using visual inspection of histograms, Q-Q plots, and residual plots along with a Shapiro-Wilk test for each trait. QTL mapping was performed using the R package “qtl” [30]. A previously developed integrated MN1264×MN1246 linkage map composed of 3024 single nucleotide polymorphism (SNP) markers was used for QTL analysis [28]. Data from 110 individuals in 2017 and 113 individuals in 2018 were used in mapping; data from nongenotyped individuals was omitted. RGB, L∗a∗b∗, HSI, and OIV values were used as phenotypic data, with each channel (red, R; green, G; blue, B; lightness, L∗; green-red, a∗; blue-yellow, b∗; hue, H; saturation, S; and intensity, I), average and weighted grey, OIV score, and noir/nonnoir mapped as separate traits. Mapping was performed using a nonparametric method [31] for all traits (due to nonnormal distributions) except blue (B), which was mapped using simple interval mapping, and noir/nonnoir, which was mapped using a binary method.

## 3. Results

In both years, segregation of fruit color (noir vs. nonnoir) was not significantly different than the expected 1 : 1 as assessed by a chi-square test (Table 1). Colors observed ranged from green-yellow to dark blue; five of the six OIV fruit color classes were observed in 2017, while only three classes were observed in 2018 (Table 1).

**Figure 4:**
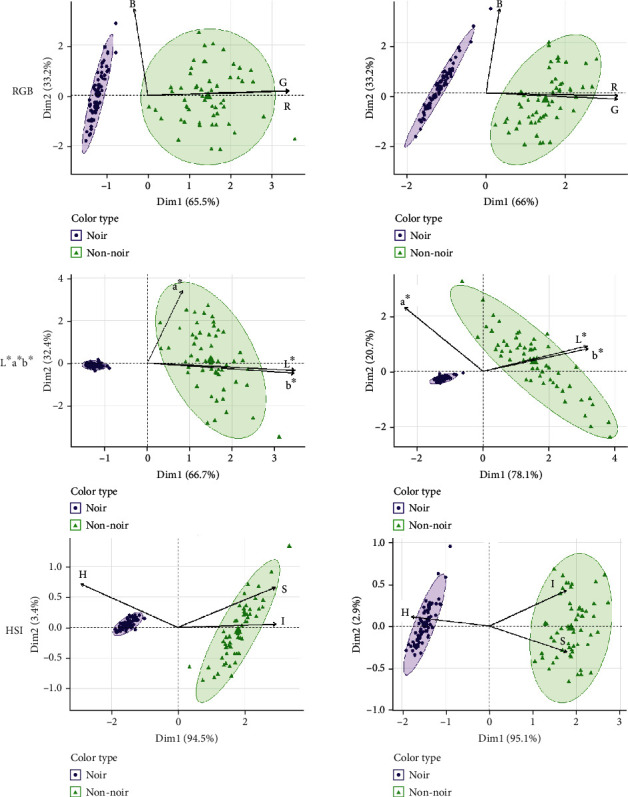
PCA biplot for RGB color in 2017 and 2018. Arrows indicate traits while circles and triangles represent each individual, coded by color.

**Figure 5:**
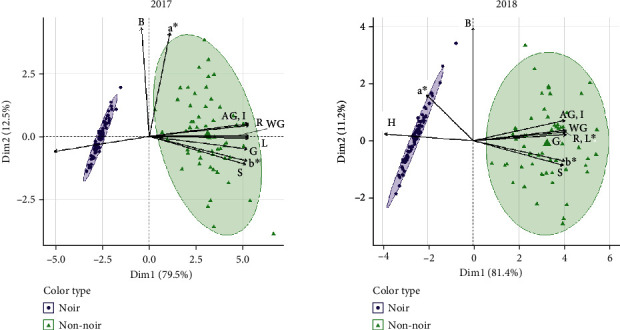
Combined PCA biplot for RGB, L∗a∗b∗, and HSI color in 2017 and 2018. Arrows indicate traits while circles and triangles represent each individual, coded by color.

Each of the three color spaces used (RGB, L∗a∗b∗, or HSI) separates colors into three different constituents. R and G values were distributed bimodally, both with highly significant Shapiro-Wilk test results in both years (*p* < 0.001; indicates a nonnormal distribution) while B values were distributed normally (*p* = 0.8355 in 2017 and *p* = 0.1565 in 2018). L∗ and b∗ values were bimodally distributed while a∗ was not; all three values were distributed nonnormally in both years (*p* < 0.001). H, S, and I were all distributed bimodally in both years and were significantly nonnormal (*p* < 0.001). When PCA was performed and genotypes plotted and classified as noir or nonnoir, tight clustering of the noir individuals can be seen in each (Figure 4). In the RGB color space, noir individuals are separated from nonnoir individuals by the first principal component, and nonnoir individuals are widely distributed. L∗a∗b∗ shows even tighter clustering of noir fruit and nonnoir fruit spread across most of the second principal component. HSI shows a distinct difference in noir individual clustering between years, with both color types showing more compact clustering in 2017 as compared to 2018. The biplot showing the PCA with all color traits combined shows the variables that most distinctly separate noir and nonnoir individuals: H, B, and a∗ are more closely associated with noir fruit, while all other variables are associated with nonnoir fruit (Figure 5).

Differences in color between years were explored using correlation tests. When considering all fruit in the GE1025 population, Pearson's correlation coefficients (*r*) were all significant (*p* < 0.05; Table 2). Values for all but two of the color channels were strongly correlated between the two years: with the exceptions of a∗ (*r* = 0.24) and B (*r* = 0.62), correlation coefficients ranged from 0.92 to 1.00. However, these correlations were lower when only looking at the nonnoir subset of the population, with correlations ranging from 0.28 (OIV score) to 0.71 (B). Correlations were even lower when considering the noir segment of the populations, with two nonsignificant correlations (green-red and hue) and significant correlations ranging from 0.37 to 0.57. Overall, these correlations indicate that some measurements of color were highly variable between years when considering both noir and nonnoir fruit.

In 2017, four distinct color QTL were significant at the genome level on chromosomes 2, 6, 10, and 15 when mapping was performed in the whole population (Table 3). The QTL detected on chromosome 2 of the maternal (MN1264) and paternal (MN1246) maps was significantly associated with 12 of the 13 mapped traits. The chromosome 2 QTL was most frequently mapped to marker S2_6921406 at 63 cM on the maternal map but also mapped to other markers from 60.1 cM (S2_5825563) to 82.0 cM (S2_9126568). On the paternal map, the QTL was found at 23 cM (S2_5352505). Variation explained by this QTL ranged from 30.79% to 93.72%, with the paternal QTL accounting for a smaller proportion of variation. On a chromosome-wide level, QTL were found on chromosome 1 of the maternal map and on chromosomes 2, 7, and 10 of the paternal map (Table 4). The variance explained by these QTL ranged from 5.53% to 29.09%.

In 2018, genome-wide significant QTL were only seen on chromosomes 2 and 6. No QTL associated with the paternal map were observed, and variation explained by each QTL ranged from 12.10% to 92.20%. At a chromosome-wide significance level, QTL were found on chromosomes 1 and 18 of the maternal map and chromosomes 2 and 15 of the paternal map. These QTL explained between 7.26% and 23.65% of variation. Only two QTL significant at a chromosome-wide level from 2017 were observed again in 2018: those associated with saturation (S) on chromosome 1 of the maternal map and OIV on chromosome 2 of the paternal map.

LOD plots for each reported QTL can be seen in Figures S1-S4.

## 4. Discussion

A major challenge in phenotyping grape berry color is the classification of berries into discrete classes despite their subtle inter- and intracluster variations. Unique microclimates resulting in different exposures to temperature and light, plus differences in individual developmental processes, lead to slight color differences which are difficult to quantify. To more fully capture this variation, image data was analyzed using several color spaces and values were used to map possible QTL influencing color in a diverse segregating F1 population. MN1264, the maternal parent, produces noir fruit and is donating at least one functional allele at each of the two *VvMYBA* loci to its progeny. The paternal parent, MN1246, produces red-skinned fruit and must be heterozygous at at least one of the color-determining loci. The genotypes segregated in a 1 : 1 ratio for noir to nonnoir, which supports the two-gene model of color currently in use. The distributions of color values also reflected these two major classes of color; the bimodal distributions represented noir and nonnoir fruit. Each color space was adept at separating noir fruit from nonnoir fruit, as evidenced by PCA. However, each space performed differently when it came to separating fruit within each group. The color spaces RGB and HSI both showed clustering of noir and nonnoir fruit, and both separated fruit within the groups using two principal components. Noir fruit showed more variation in 2018 than in 2017 when using the HSI color space, while RGB performed similarly in both years. L∗a∗b∗ was not able to separate noir fruit due to homogeneous a∗ and b∗ values between fruit, making it the least suitable color space for berry color classification.

For nearly every visual color trait measured, a strong QTL from the maternal parent was detected on chromosome 2. Each likely colocalizes with the *VvMYBA1* or *VvMYBA2* gene(s), those responsible for determination of noir or nonnoir fruit color. The most frequent site of a logarithm of odds (LOD) value maximum was a marker located at 63 cM, with 1.5 LOD confidence intervals ranging from 51 cM to 87 cM. In 2017, a QTL for red (R) on the paternal map was observed with a confidence interval that overlapped with the *V. vinifera LDOX* gene region [6]. QTL significant on a chromosome-wide level identified regions of interest where multiple color traits had high LOD values, such as marker S2_5352505 at 23 cM on chromosome 2 of the paternal parent (MN1246) map or marker S1_12013218 near the beginning of chromosome 1 on the maternal parent (MN1264) map. Several of these major and minor QTL have been reported in a study of anthocyanin-related traits performed by Costantini et al. [24], where significance was also assessed on a chromosome-wide basis. Though anthocyanin and color are not synonymous, loci reported in these results overlapped with those reported in [24]. This includes the loci on maternal chromosomes 2 and 6 and paternal chromosomes 2 and 10 on a genome-wide level and on maternal chromosome 1 and paternal chromosome 2 on a chromosome-wide level. The apparent relationship between observed color and measured anthocyanin suggests that image-based phenotyping techniques are successful in identifying loci of interest. Differences between years were seen in the QTL results: a QTL for blue (B) on chromosome 15 and one for green-red (a∗) on chromosome 10 were detected in 2017 but not in 2018. This was likely due to the greater variation observed in visual color in 2017, where a greater number of color classes were seen. Additionally, differences were seen in the results for putative QTL significant at a chromosome-wide level; only two of the 18 observed QTL occurred in both years. It is likely that environmental differences caused by weather during véraison and ripening contributed to both of these variations, as August 2017 (véraison) was cooler and wetter than August 2018 (Table S1). Low temperatures increase anthocyanin accumulation, so this may be one explanation for the greater number of rose, red, and grey nonnoir individuals in 2017. The difference in QTL observed between years is evidence for genotype-by-environment interaction occurring for this trait, and continuing to study berry color over a number of years could help elucidate its impact. However, the repeated emergence of these two QTL despite environmental variation indicates their status as loci of interest. It is likely that the use of quantitative color measurements, rather than qualitative categorization, helped in detecting these minor QTL.

**Figure 6:**
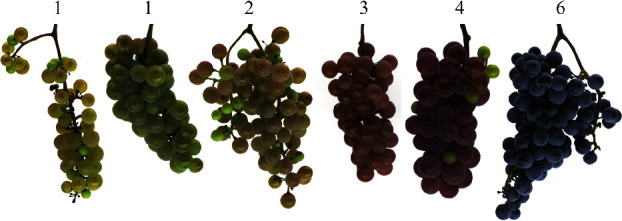
A wide range of berry colors was observed in GE1025, with clusters ranging from yellow-green (left) to blue black (right). OIV descriptor 225 score is listed above each cluster.

**Figure 7:**
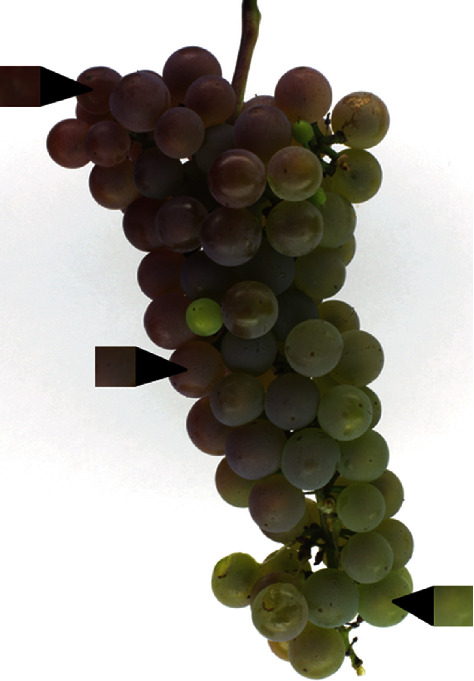
Examples of color variability from within a single cluster.

One unique aspect of this mapping population was the range of its color diversity (Figure 6). A wide range of colors was observed in both years, which was ideal for identifying smaller-effect loci along with the previously known major QTL. The diversity of colors observed among nonnoir fruit reflected their differing anthocyanin profiles. Some were yellow-green (OIV descriptor 225, rating of 1) and had a lower anthocyanin content, some were pink-to-red (OIV descriptor 225, ratings of 2, 3, and 4) with higher anthocyanin content, and some showed variability among and within berries, as skin color differed depending on sun exposure (Figure 7). This is represented in the PCA, where each visual color space is able to show separation between nonnoir individuals. Environmental effects on anthocyanin synthesis have been well-documented, with temperature [12], sun or shade exposure [13], and plant hormones [14, 15] all contributing to accumulation or degradation of anthocyanins. This is in addition to genetic effects, including the large-effect *VvMYBA1* and *VvMYBA2* loci, and loci of smaller effect identified elsewhere in the genome. Genotype-by-environment interactions are also likely, with nonnoir individuals displaying differently colored fruit between different environmental conditions as demonstrated between the two years. All of these likely influenced and resulted in fruit that exhibited considerable variation, even between clusters on the same plant or berries within the same cluster. Measuring color using numeric values helped alleviate this issue by having the capacity to quantify differences between fruit that would otherwise fall into the same category of visual color. For example, fruit described as “rose” could vary in the degree to which they were red or green, while the automated method was able to quantify that variation using different color space values. A challenge that still remains is the way to best deal with berries that exhibit multiple colors. In our pipeline and analysis, colors were defined by the average color values for pixels classified as “berry” in a segmented grape cluster image. In the future, fruit could also be rated by its color extremes (most blue/red area, most green area) or by the pixel color that occurred most frequently within berries in order to compare variation within and between genotypes.

An advantage of our phenotyping platform is its low cost and ease of use. For each image, a user only has to select one to a few areas of pixels within berries to return the average color of an entire cluster. The equipment required (camera, tripod, and lighting) is inexpensive and can be set up in many environments, with the ability to move if needed: for example, acquiring images near where the fruit is harvested, as opposed to transporting the fruit back to a lab for imaging. Although information about berry chemical composition cannot necessarily be collected using image analysis, further research investigating the relationship between anthocyanins and visual color could identify whether chemistry information could be inferred. Similarly, spectrophotometric or hyperspectral data in conjunction with images could aid in understanding whether specific compounds contributed to or affected visual color. As mentioned previously, the wide range of color observed in GE1025 lent to the ability to identify minor QTL. Particularly, an advantage of evaluating such a diverse population is the potential for marker development for use in marker-assisted selection. Rather than selecting for simply noir or nonnoir fruit, markers could be developed to select for particular hues. Visual color is a high-value trait in both wine and table grapes, and continued research from different angles will help give a fuller picture of the complex mechanisms behind its control. The potential to use marker-assisted selection for specific colors in a grape-breeding program would increase efficiency, with the predicted progeny performance (i.e., color) at the seedling stage instead of after fruits are produced. This could save years of time, effort, and vineyard space, along with the cost associated with each.

This study used a semiautomated image segmentation process to quantify berry color in a population of diverse hybrid wine grapes. Color data isolated from images was used to confirm major color QTL and identified new minor QTL affecting color. Using several different color spaces allowed variation to be explored in multiple ways, and L∗a∗b∗ was found to be the least suitable color space for differentiating within the noir and nonnoir color classes. Continuing to explore visual color, including its relationship with anthocyanins and other chemical compounds, will help increase the understanding of the environmental and genetic effects on this economically important trait.

## Figures and Tables

**Table 1:** GE1025 population categorized by OIV color score in 2017 and 2018, along with *p* value for chi-square analysis of a 1 : 1 segregation ratio between noir and nonnoir individuals for each year. “Noir” refers to blue-black (6) color, while “nonnoir” includes all others (1-5).

Color (OIV score)	2017 (*n* = 121)	2018 (*n* = 127)
Green-yellow (1)	11	23
Rose (2)	40	33
Red (3)	3	0
Grey (4)	1	0
Dark red violet (5)	0	0
Blue black (6)	66	71
Noir individuals	66	71
Nonnoir individuals	55	56
Chi-square *p* value	0.317	0.183

**Table 2:** Pearson correlation coefficients (*r*) for color components between years (*p* < 0.05 in all cases). “All” is indicative of the correlation including all individuals, while “nonnoir” and “noir” only examined those color subsets of the population. Dashes indicate lack of significant correlation.

Color component	*r* (all; *n* = 117)	*r* (nonnoir; *n* = 53)	*r* (noir; *n* = 64)
Red (R)	0.97	0.51	0.44
Green (G)	0.92	0.46	0.47
Blue (B)	0.62	0.71	0.51
Lightness (L∗)	0.94	0.49	0.47
Red-green (a∗)	0.20	0.40	—
Blue-yellow (b∗)	0.96	0.55	0.40
Hue (H)	1.00	0.36	—
Saturation (S)	0.95	0.60	0.30
Intensity (I)	0.94	0.52	0.48
Weighted grey	0.94	0.49	0.47
Average grey	0.94	0.52	0.48
OIV score	0.98	0.28	—

**Table 3:** QTL significant at a genome-wide level using a threshold of *α* = 0.05 for image-derived color values in the GE1025 population (2017: *n* = 110, 2018: *n* = 113). Parental map specifies the parent from which the QTL originates (maternal: MN1264; paternal: MN1246).

Year	Trait	Chr	Parental map	Max LOD	Closest marker	Position (cM)	1.5 LOD interval (cM)	Variance explained (%)
2017	Red (R)	2	Maternal	52.9	S2_9126568	81.0	61.0-87.0	89.08
		2	Paternal	8.79	S2_5352505	23.0	11.0-27.0	30.79
	Green (G)	2	Maternal	52.7	S2_6921406	63.0	61.0-85.0	88.99
		2	Paternal	9.24	S2_5352505	23.0	17.0-25.4	32.08
	Blue (B)	6	Maternal	4.66	S6_6834834	33.0	0.0-40.0	15.78
		15	Maternal	4.80	S15_18026522	93.7	89.0-111.9	16.21
	Lightness (L∗)	2	Maternal	52.8	S2_6921406	63.0	61.0-86.0	89.04
		2	Paternal	8.98	S2_5352505	23.0	17.0-25.4	31.34
	Green-red (a∗)	2	Maternal	17.6	S2_5825563	60.1	59.3-86.0	52.14
		10	Paternal	9.03	S10_2470325	1.0	0.0-25.0	31.48
	Blue-yellow (b∗)	2	Maternal	53.3	S2_9126568	82.0	62.0-88.0	89.26
	Hue (H)	2	Maternal	52.9	S2_6921406	63.0	61.0-82.0	89.08
	Saturation (S)	2	Maternal	52.9	S2_6921406	63.0	59.0-82.0	89.08
	Intensity (I)	2	Maternal	52.8	S2_6921406	63.0	61.0-85.0	89.04
	Weighted grey	2	Maternal	52.8	S2_6921406	63.0	61.0-86.0	89.04
		2	Paternal	8.8	S2_5352505	23.0	17.0-25.4	30.82
	Average grey	2	Maternal	52.8	S2_6921406	63.0	61.0-85.0	89.04
	OIV	2	Maternal	66.1	S2_7271746	63.4	61.0-84.0	93.72
	Noir/nonnoir	2	Maternal	32.8	S2_9126568	79.0	61.0-82.0	74.67

2018	Red (R)	2	Maternal	50.5	S2_9126568	79.0	78.0-83.0	87.23
	Green (G)	2	Maternal	50.6	S2_9126568	79.0	78.0-83.0	87.28
	Blue (B)	6	Maternal	3.16	S6_2546367	5.0	0.0-37.0	12.10
	Lightness (L∗)	2	Maternal	50.6	S2_9126568	81.0	78.0-83.0	87.28
	Green-red (a∗)	2	Maternal	9.5	S2_5825563	60.1	58.0-82.0	32.02
	Blue-yellow (b∗)	2	Maternal	49.0	S2_9126568	81.0	62.0-84.0	86.42
	Hue (H)	2	Maternal	50.3	S2_9126568	81.0	64.2-83.0	87.13
	Saturation (S)	2	Maternal	51.1	S2_9126568	81.0	61.0-87.0	87.54
	Intensity (I)	2	Maternal	50.9	S2_9126568	81.0	78.0-83.0	87.44
	Weighted grey	2	Maternal	50.6	S2_9126568	81.0	78.0-83.0	87.28
	Average grey	2	Maternal	50.9	S2_9126568	81.0	78.0-83.0	87.44
	OIV	2	Maternal	62.6	S2_9126568	81.0	78.0-84.0	92.20
	Noir/nonnoir	2	Maternal	30.0	S2_9126568	79.0	78.0-83.0	70.55

**Table 4:** QTL significant at a chromosome-wide level using a threshold of *α* = 0.05 for image-derived color values in the GE1025 population (2017: *n* = 110, 2018: *n* = 113). Parental map specifies the parent from which the QTL originates (maternal: MN1264; paternal: MN1246).

Year	Trait	Chr	Parental map	Max LOD	Closest marker	Position (cM)	Variance explained (%)
2017	Red (R)	1	Maternal	3.63	S1_12013218	0.9	13.75
	Green (G)	1	Maternal	3.62	S1_12013218	0.9	13.72
	Blue-yellow (b∗)	1	Maternal	5.88	S1_12013218	0.9	21.31
	Hue (H)	7	Paternal	4.98	S7_1435517	2.7	18.37
		10	Paternal	5.80	S10_2470325	0.0	21.05
	Saturation (S)	1	Maternal	3.64	S1_22128396	19.7	13.79
	Intensity (I)	2	Paternal	8.21	S2_5352505	23.0	28.44
	Average grey	2	Paternal	8.18	S2_5352505	23.0	28.35
	OIV	2	Paternal	4.68	S2_5352505	23.0	17.36
	Noir/nonnoir	1	Maternal	1.36	S1_12013218	0.9	5.39

2018	Green (G)	2	Paternal	4.43	S2_5352505	23.0	16.52
	Green-red (a∗)	15	Paternal	5.76	S15_12895291	39.8	20.92
		18	Maternal	6.62	S18_2451141	5.4	23.65
	Hue (H)	1	Maternal	4.22	S1_22128396	19.7	15.80
	Saturation (S)	1	Maternal	4.49	S1_23506714	24.0	16.72
		2	Paternal	4.69	S2_5352505	23.0	17.40
	OIV	2	Paternal	5.47	S2_5352505	23.0	19.98
	Noir/nonnoir	2	Paternal	1.85	S2_5352505	23.0	7.26

## Data Availability

All images are stored in the Data Repository for the University of Minnesota (DRUM) and are available in entirety upon request at http://hdl.handle.net/11299/202560. The image processing MATLAB script is publicly available at https://www.github.com/underhillanna/GrapeImageAnalysis.

## Supplementary Materials

Supplementary Materials Table S1: mean August weather data in Excelsior, MN (44°52′08.1^″^N 93°38′17.3^″^W; weather station KMNEXCEL9) for 2017 and 2018. High, average, and low temperatures are daily means; daily precipitation is a monthly average. Figures S1–S4: see attached material; LOD plots corresponding to Tables 3 and 4 for QTL significant at genome-wide and chromosome-wide levels.
