# Supplementary material for: Evaluating and Mapping Grape Color Using Image-Based Phenotyping
Source: Plant Phenomics. 2020 Apr 24;2020:8086309. doi: 10.34133/2020/8086309 (PMC7706331; doi:10.34133/2020/8086309)
Supplement: Supplementary Materials — Table S1: mean August weather data in Excelsior, MN (44°52′08.1″N 93°38′17.3″W; weather station KMNEXCEL9) for 2017 and 2018. High, average, and low temperatures are daily means; daily precipitation is a monthly average. Figures S1–S4: see attached material; LOD plots corresponding to Tables 3 and 4 for QTL significant at genome-wide and chromosome-wide levels. [file 8086309.f1.docx]

**Supplementary Materials**

**Figure S1.** Logarithm of odds (LOD) score plots for QTL significant on a genomewide level in 2017. Red solid lines indicate LODs and dashed black lines indicate significance thresholds (α = 0.05). X-axis is location on the chromosome in centimorgans (cM), and perpendicular lines on x-axis indicate marker locations.


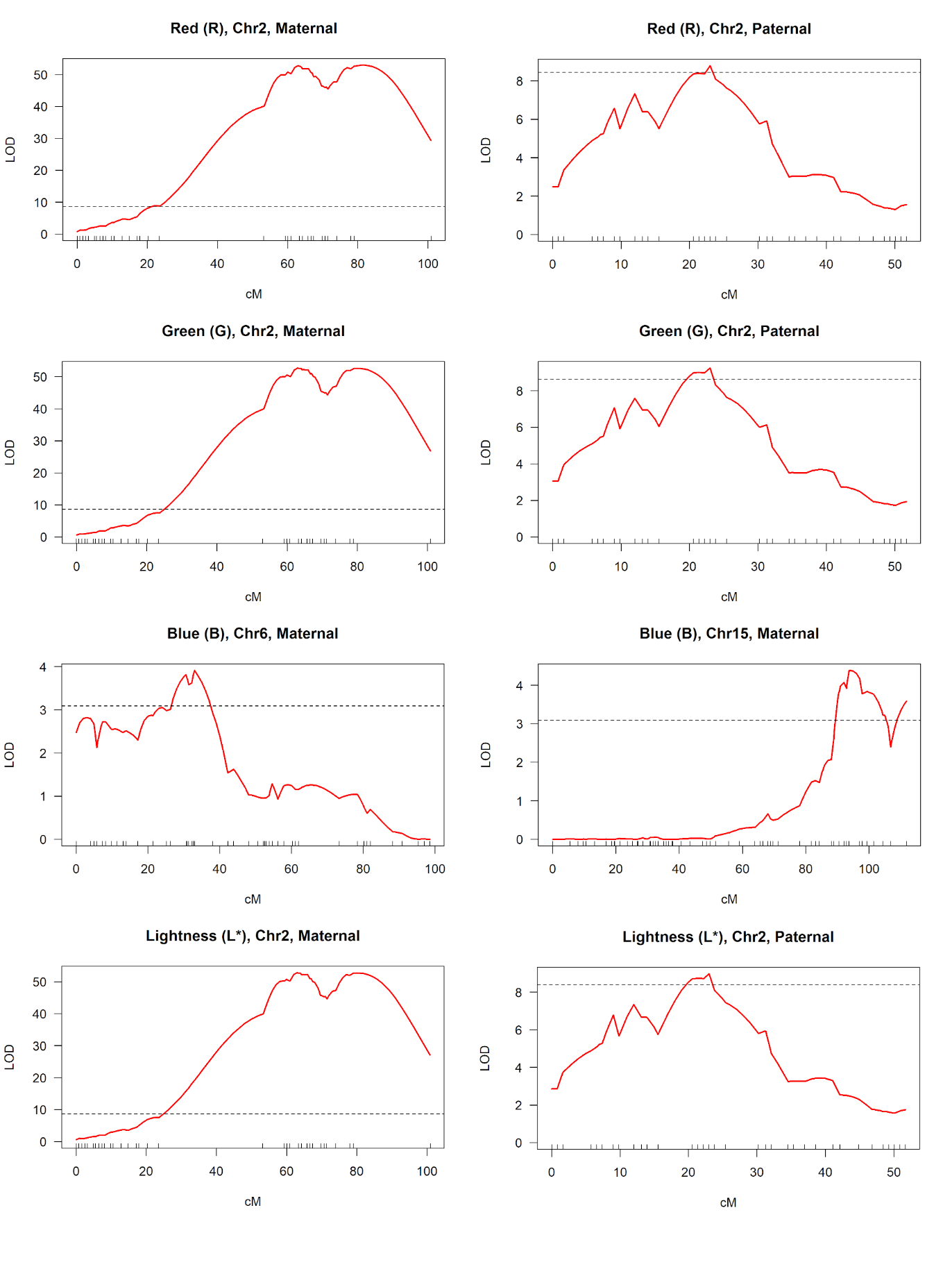


**Figure S1**, continued


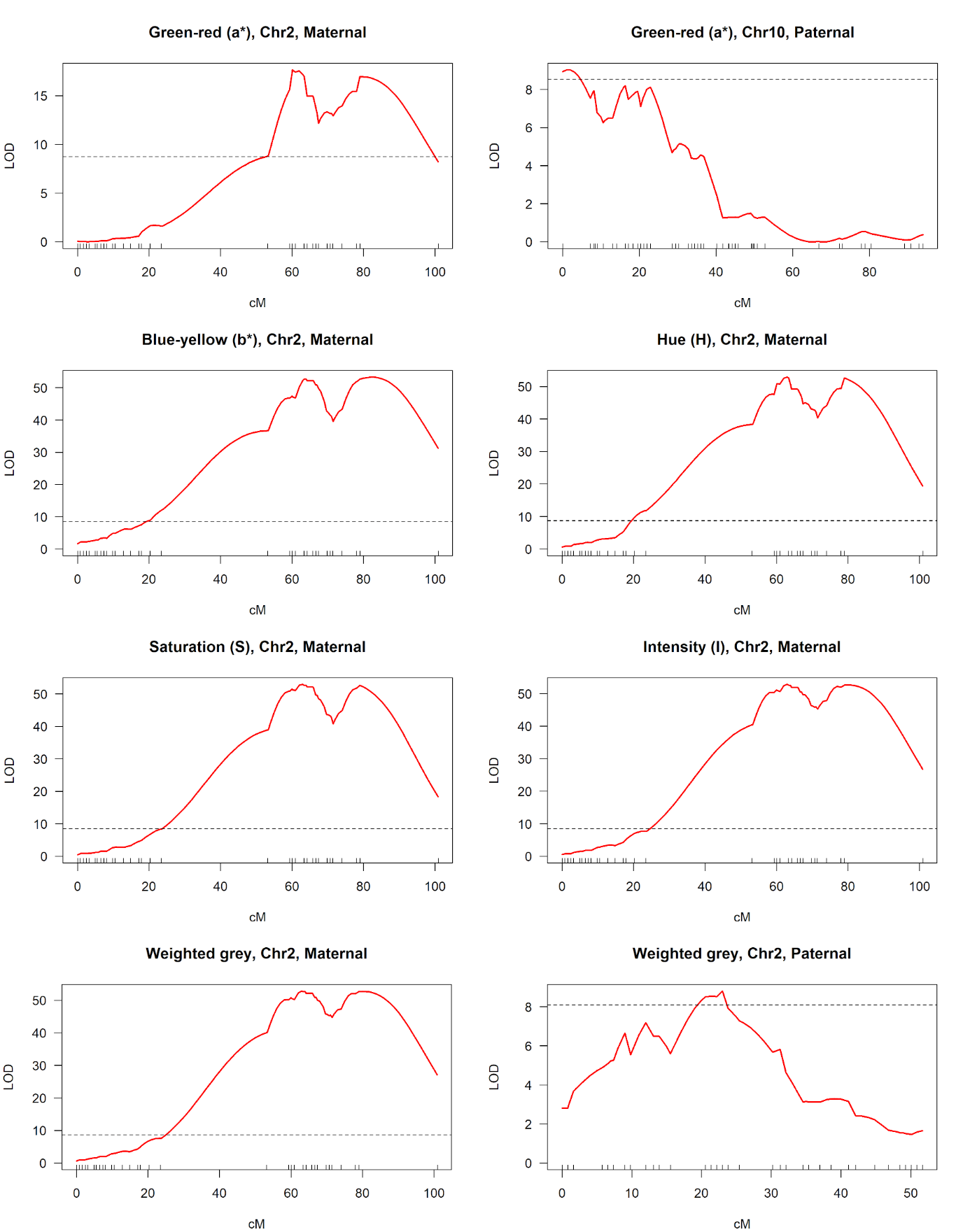


**Figure S1**, continued


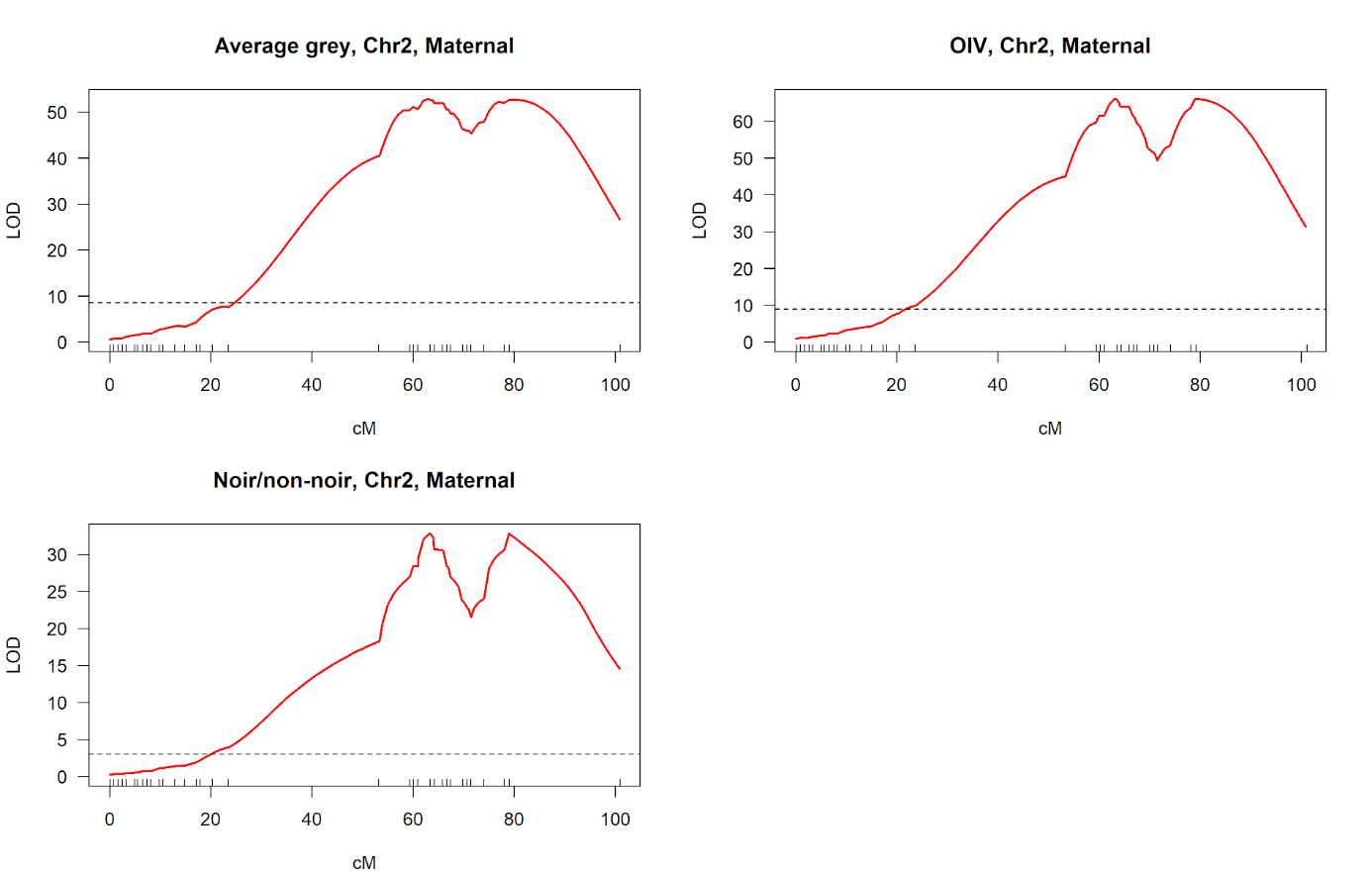


**Figure S2.** Logarithm of odds (LOD) score plots for QTL significant on a genomewide level in 2018. Red solid lines indicate LODs and dashed black lines indicate significance thresholds (α = 0.05). X-axis is location on the chromosome in centimorgans (cM), and perpendicular lines on x-axis indicate marker locations.


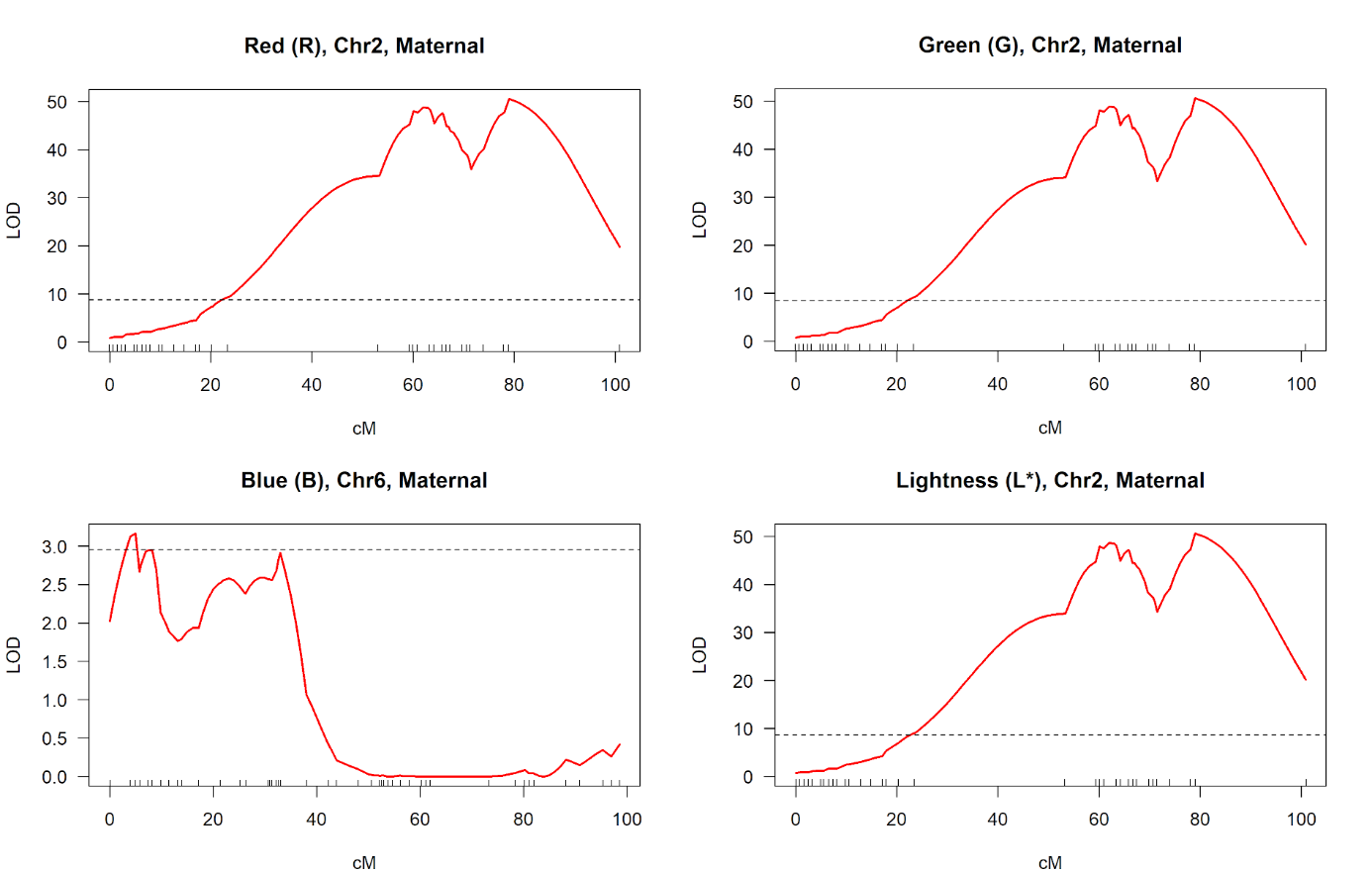


**Figure S2**, continued


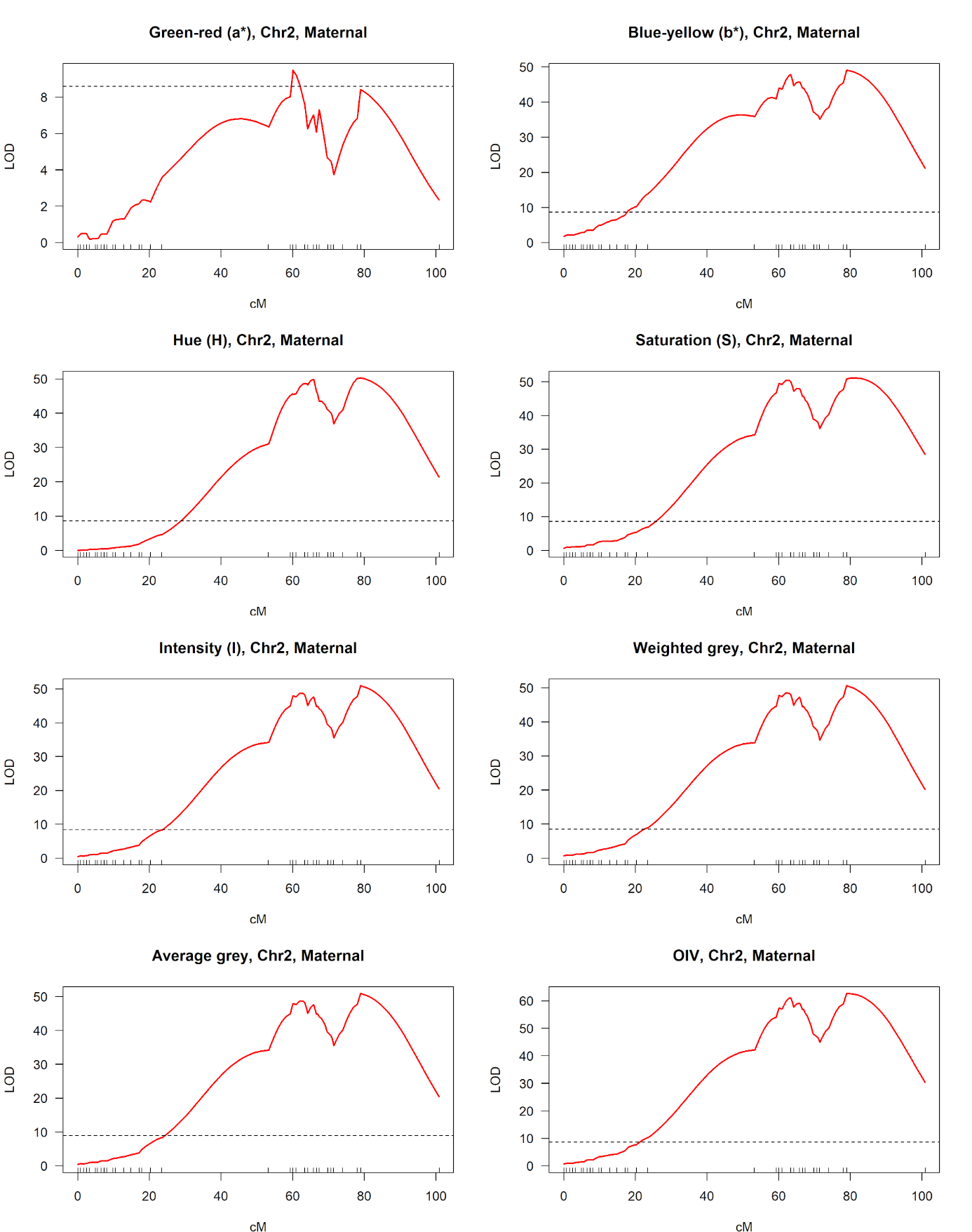


**Figure S2,** continued


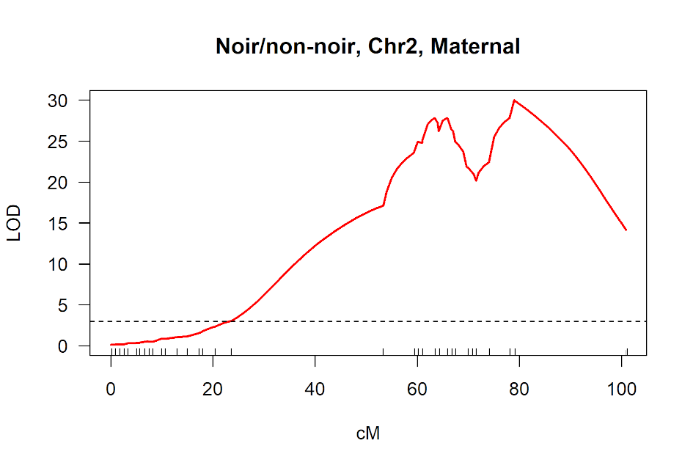


**Figure S3.** Logarithm of odds (LOD) score plots for QTL significant on a chromosome-wide level in 2017. Red solid lines indicate LODs and dashed black lines indicate significance thresholds (α = 0.05). X-axis is location on the chromosome in centimorgans (cM), and perpendicular lines on x-axis indicate marker locations.


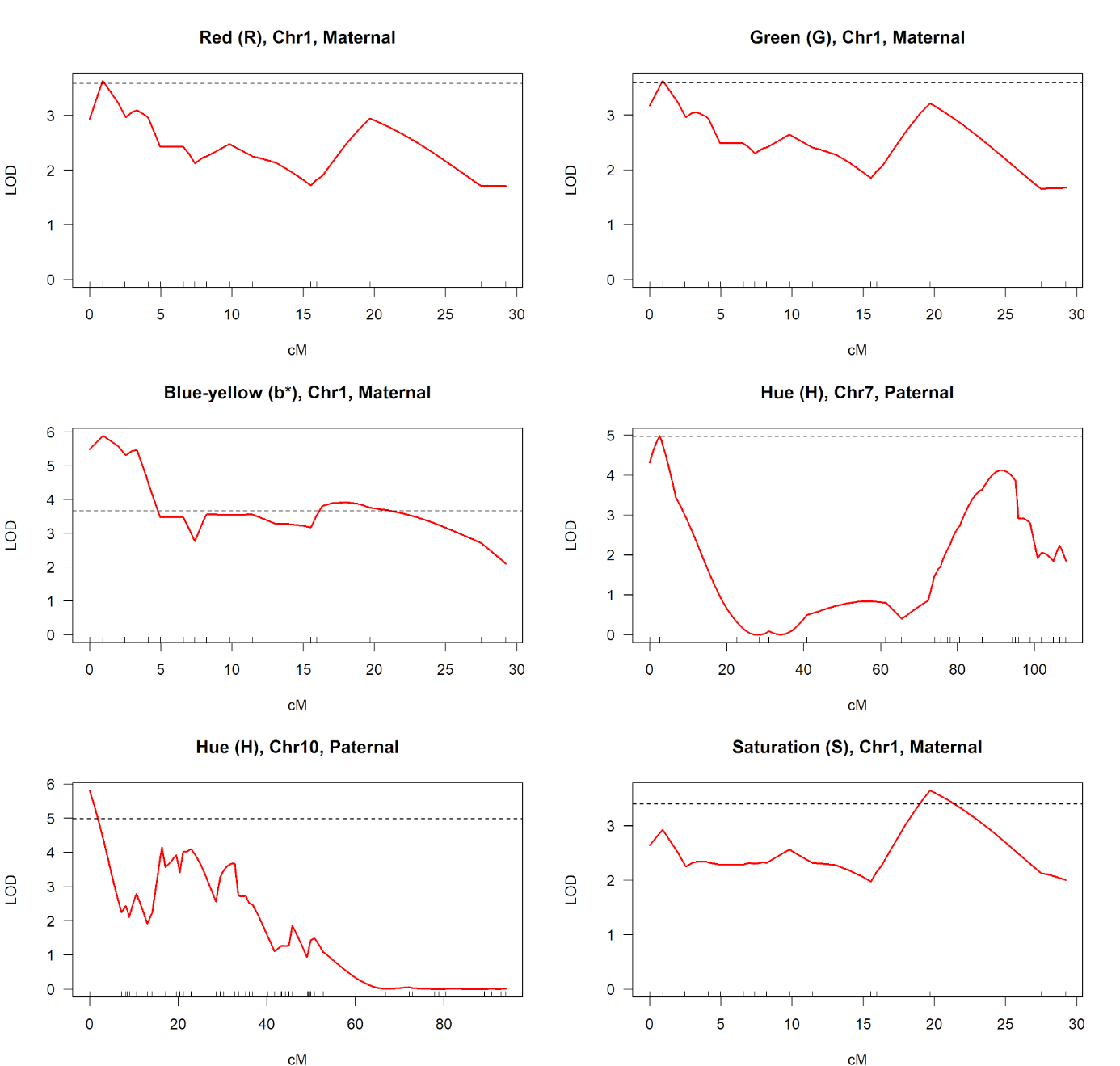


**Figure S3**, continued


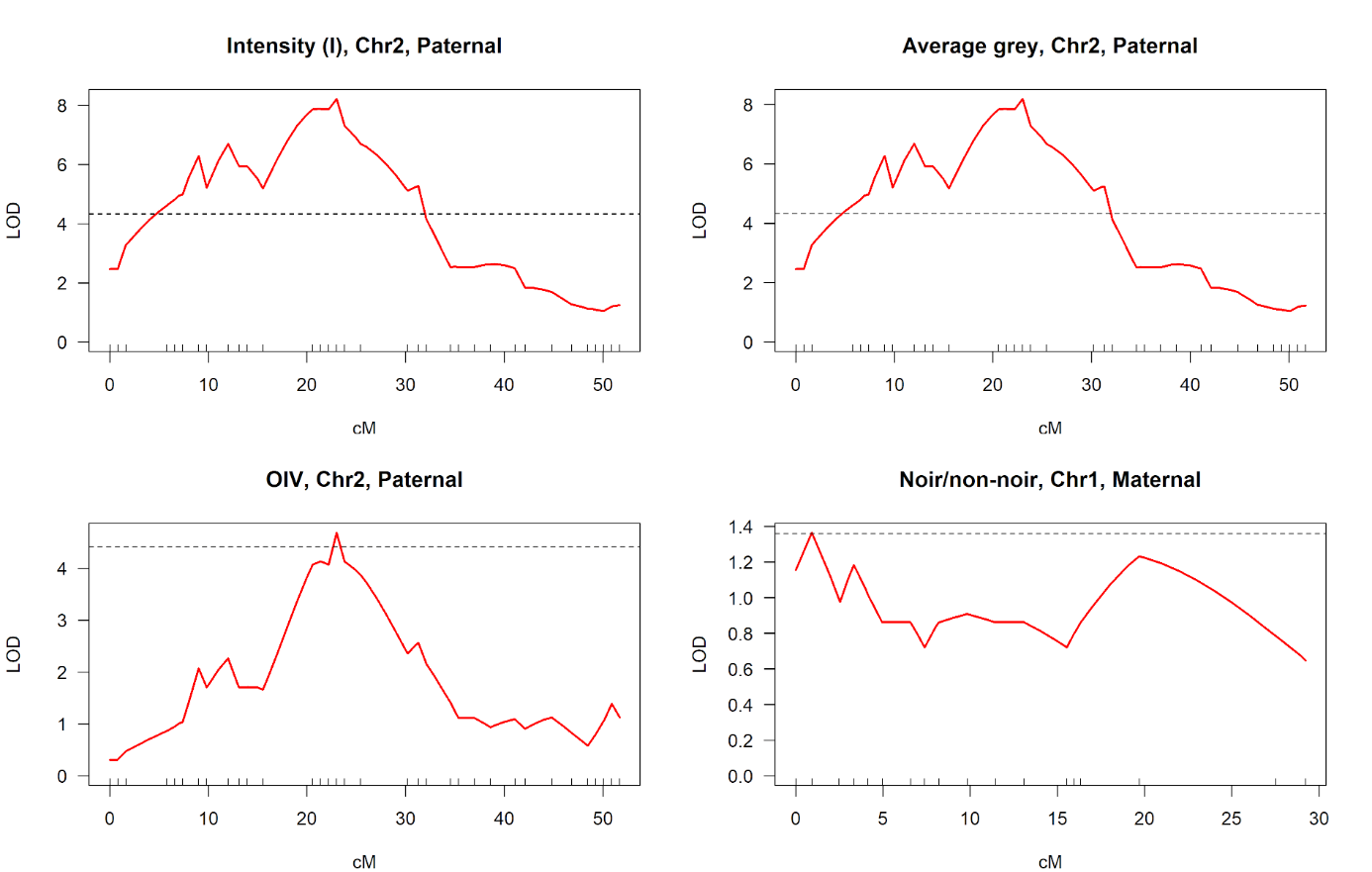


**Figure S4.** Logarithm of odds (LOD) score plots for QTL significant on a chromosome-wide level in 2018. Red solid lines indicate LODs and dashed black lines indicate significance thresholds (α = 0.05). X-axis is location on the chromosome in centimorgans (cM), and perpendicular lines on x-axis indicate marker locations.


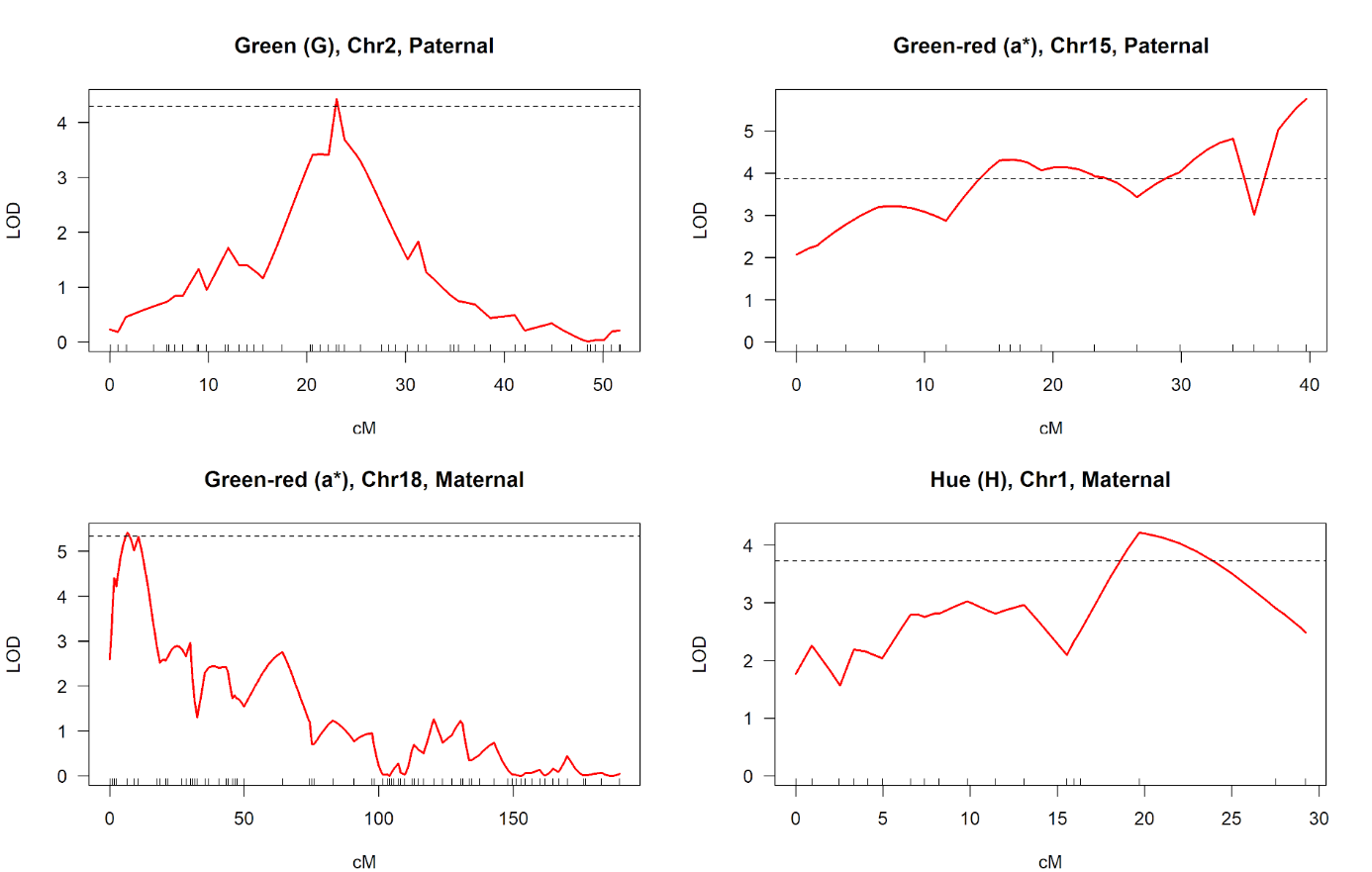


**Figure S4**, continued


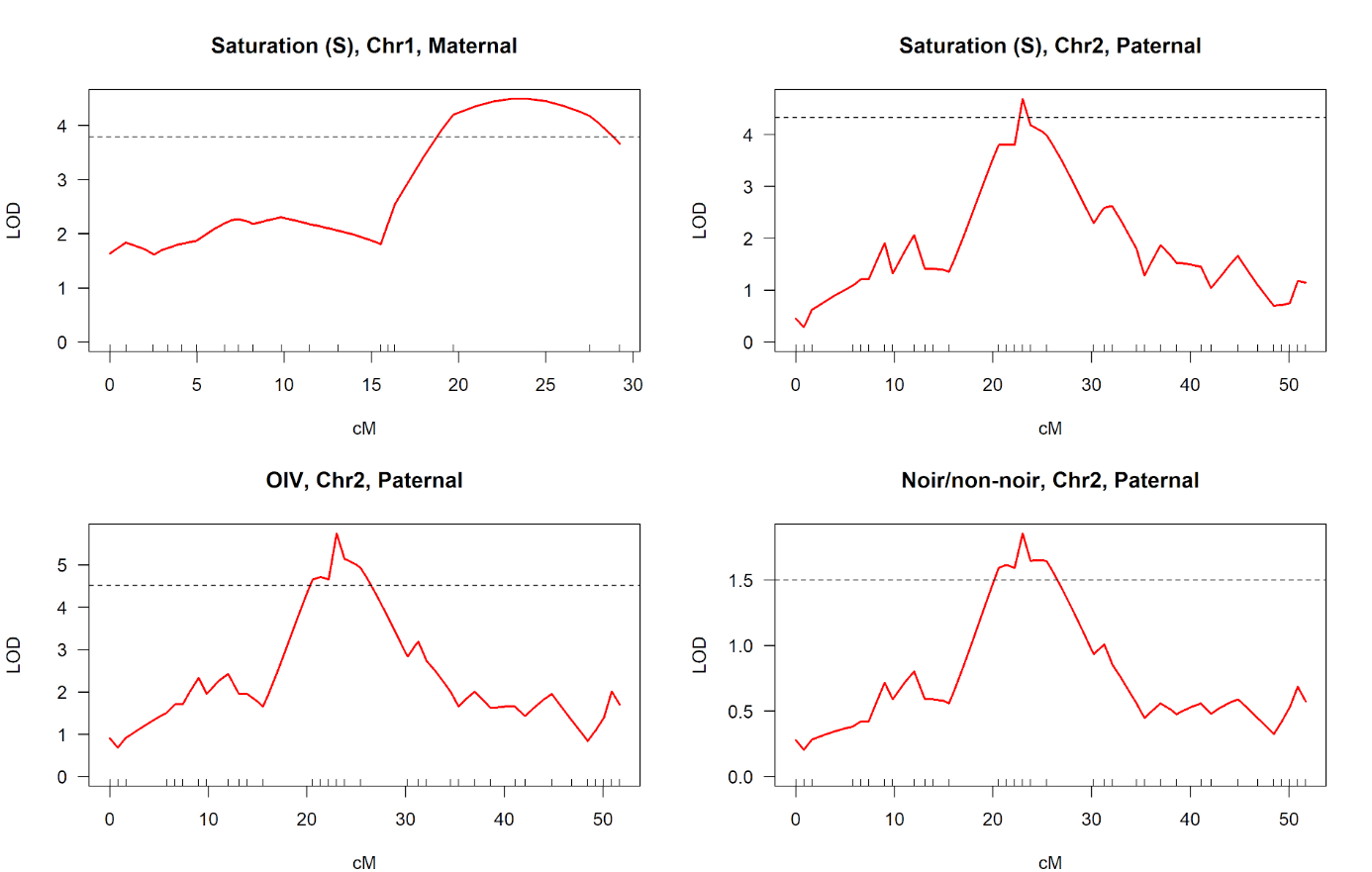


**Table S1.** Mean August weather data in Excelsior, MN (44°52’08.1”N 93°38’17.3”W; weather station KMNEXCEL9) for 2017 and 2018. High, average, and low temperatures are daily means; daily precipitation is a monthly average.

| Year | High (°C) | Average (°C) | Low (°C) | Daily precipitation (mm) | Days > 26 °C | Days < 18 °C | Days with precipitation |
| --- | --- | --- | --- | --- | --- | --- | --- |
| 2017 | 25.14° | 20.34° | 15.88° | 5.33 | 10 | 27 | 14 |
| 2018 | 27.67° | 22.31° | 17.38° | 1.78 | 21 | 19 | 9 |
